# 2020 Mid-Atlantic Telehealth Resource Center Annual Summit

**DOI:** 10.5195/ijt.2019.6294

**Published:** 2019-12-12

**Authors:** Anita Browning

**Figure:**
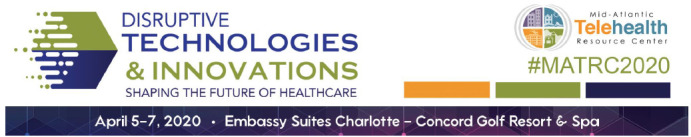


The Mid-Atlantic Resource Center (MATRC; http://www.matrc.org/) advances the adoption and utilization of telehealth within the MATRC region and works collaboratively with the other federally funded Telehealth Resource Centers to accomplish the same nationally. MATRC offers technical assistance and other resources within the following mid-Atlantic states: Delaware, District of Columbia, Kentucky, Maryland, North Carolina, New Jersey, Pennsylvania, Virginia and West Virginia.

The 2020 MATRC Summit will be held April 5-7, 2020, at The Embassy Suites Charlotte–Concord Golf Resort and Spa, Concord, NC. Attendees need not reside in a mid-Atlantic state. This year's Summit will explore how disruptive technologies and innovations are shaping the future of healthcare. The inter-disciplinary program will feature pre-Summit educational sessions, keynotes, panels, “research flash” presentations, state meetings, poster sessions, a hackathon, a telehealth technology showcase, and other exhibits.

For further information and registration, visit: http://matrcsummit.org/registration.html

Discounted early bird registration will close January 31, 2020.

